# Supplementary material for: Fully-automated and ultra-fast cell-type identification using specific marker combinations from single-cell transcriptomic data
Source: Nat Commun. 2022 Mar 10;13:1246. doi: 10.1038/s41467-022-28803-w (PMC8913782; doi:10.1038/s41467-022-28803-w)
Supplement: Supplementary file 1 — Supplementary Information [file 41467_2022_28803_MOESM1_ESM.pdf]

SUPPLEMENTARY FIGURES

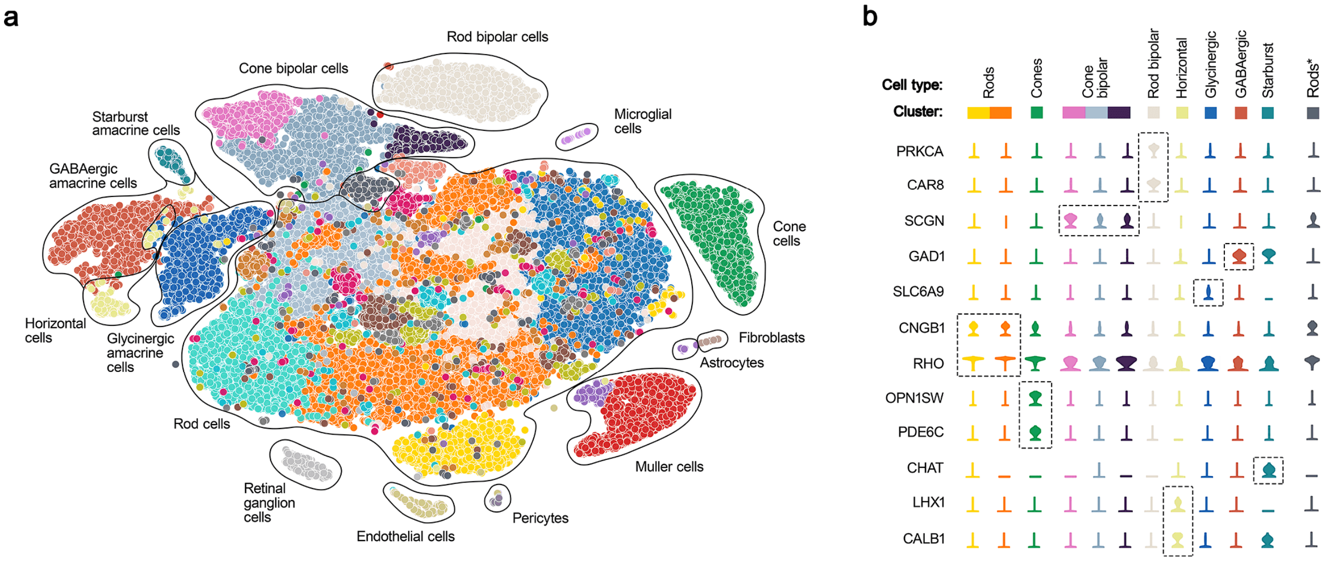

Supplementary Figure 1. ScType cell-type annotation of mouse retina scRNAseq dataset.[23] **(a)** UMAP plot shows the automated cell-type annotations with ScType. **(b)** Violin plots show the expression levels of the high-specificity marker genes that were used as a validation of correct cell-type assignments.

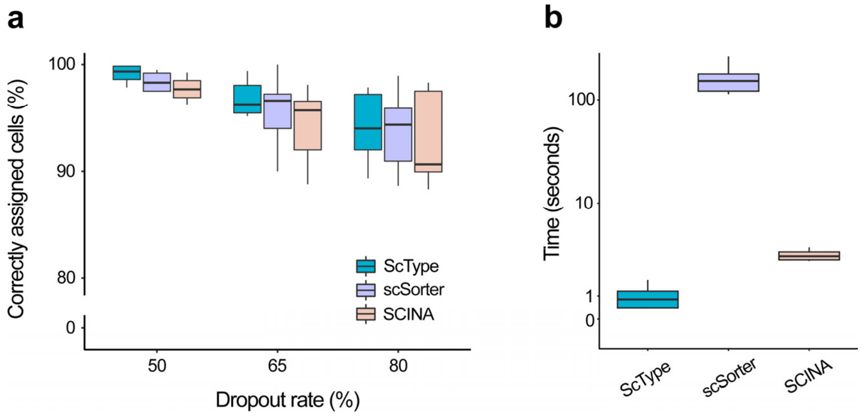

Supplementary Figure 2. Comparison of ScType, scSorter and SCINA accuracy and runtime performance. (a) The cell type annotation accuracy was tested in 45 simulated datasets with various dropout rates. **(b)** The ScType running time was faster compared to the other methods across the 45 simulated datasets (P<0.0001, two-sided Wilcoxon test). Note: the time-axis is log-scaled. The horizontal lines of box-plots, in panels a and b, indicate the median, the boxes - interquartile range (IQR), and the whiskers are Q1 - 1.5*IQR and Q3 + 1.5*IQR (where Q1 and Q3 are firsts and third quartiles), across the 15 simulated datasets in each condition.

a

	Marker (+ve)	Marker (-ve)
Gene1	T1,T2,T3	
Gene2	T1	
Gene3	T2	
Gene4	T3	T1
Gene5	T3	
Gene6	T2,T3	

	Occurence
Gene1	3
Gene2	1
Gene3	1
Gene4	2
Gene5	1
Gene6	2

	Specificity score
Gene1	0
Gene2	1
Gene3	1
Gene4	0.5
Gene5	1
Gene6	0.5

b

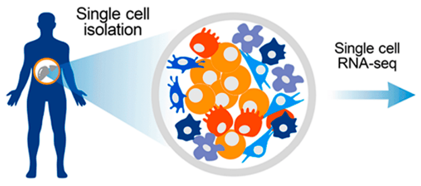

Raw expression matrix

	C1	C2	C3	C4	C5	C6
Gene1	250	230	12	210	22	240
Gene2	215	217	16	23	16	32
Gene3	19	23	300	31	33	32
Gene4	25	27	23	250	245	240
Gene5	30	32	19	0	239	233
Gene6	31	231	255	238	245	0

Z-transformed expression matrix

	C1	C2	C3	C4	C5	C6
Gene1	0.80	0.62	-1.33	0.44	-1.24	0.71
Gene2	1.28	1.30	-0.70	-0.63	-0.70	-0.54
Gene3	-0.69	-0.66	1.06	1.50	-0.60	-0.61
Gene4	-0.65	-0.63	-0.66	-0.65	1.31	1.27
Gene5	-0.56	-0.54	-0.65	-0.82	1.31	1.26
Gene6	-0.83	-0.81	0.98	0.84	0.90	-1.08

	Specificity score
Gene1	0
Gene2	1
Gene3	1
Gene4	0.5
Gene5	1
Gene6	0.5

×

Z-transformed expression matrix

	C1	C2	C3	C4	C5	C6
Gene1	0.80	0.62	-1.33	0.44	-1.24	0.71
Gene2	1.28	1.30	-0.70	-0.63	-0.70	-0.54
Gene3	-0.69	-0.66	1.06	1.50	-0.60	-0.61
Gene4	-0.65	-0.63	-0.66	-0.65	1.31	1.27
Gene5	-0.56	-0.54	-0.65	-0.82	1.31	1.26
Gene6	-0.83	-0.81	0.98	0.84	0.90	-1.08

Weighted expression matrix

	C1	C2	C3	C4	C5	C6
Gene1	0	0	0	0	0	0
Gene2	1.28	1.30	-0.70	-0.63	-0.70	-0.54
Gene3	-0.69	-0.66	1.06	1.50	-0.60	-0.61
Gene4	-0.32	-0.31	-0.33	-0.32	0.66	0.63
Gene5	-0.56	-0.54	-0.65	-0.82	1.31	1.26
Gene6	-0.42	-0.40	0.49	0.42	0.45	-0.54

Positive marker set expression

	C1	C2	C3	C4	C5	C6
MS1	0.90	0.92	-0.50	-0.45	-0.50	-0.38
MS2	-0.64	-0.62	0.90	1.11	-0.09	-0.66
MS3	-0.65	-0.63	-0.25	-0.4	1.21	0.68

-

Negative marker set expression

	C1	C2	C3	C4	C5	C6
MS1	-0.32	-0.31	-0.33	-0.32	0.66	0.63
MS2	0	0	0	0	0	0
MS3	0	0	0	0	0	0

Cell-type enrichment score

	C1	C2	C3	C4	C5	C6
MS1	1.23	1.23	-0.16	-0.12	-1.15	-1.02
MS2	-0.64	-0.62	0.90	1.11	-0.09	-0.66
MS3	-0.64	-0.63	-0.25	-0.36	1.21	0.68

Supplementary Figure 3. An example of calculating marker specificity score and ScType score. (a) In this toy example, Gene1 is a non-specific marker, and expressed in all 3 cell types (T1,T2,T3), whereas Gene2, Gene3, and Gene5 are specific markers for cell types T1, T2, and T3, respectively. Gene4 is a positive marker for cell type T3 and negative marker for cell type T1. Gene 6 is a positive marker for cell types T2 and T3. In order to calculate the marker specificity score (the right column), the marker occurrence across the cell types (middle column) is counted and scaled between 0 to 1 (0 indicates non-specific markers, maximum occurrence; and 1 for highly specific markers, minimum occurrence). **(b)** To calculate the enrichment score based on the marker expression in ScType, the raw expression data is first normalized and Z-transformed. Next, the normalized matrix is multiplied by the cell-type specificity score. Then, the expression scores of all the positive markers corresponding to a particular cell type are summarized into a single enrichment score by summing them and dividing by square root of their number. The same is done for the negative markers. Finally, the negative marker expression score is subtracted from the positive score to obtain the final enrichment score. Individual cells are assigned to a cell type based on the maximum value for the cell type marker set.

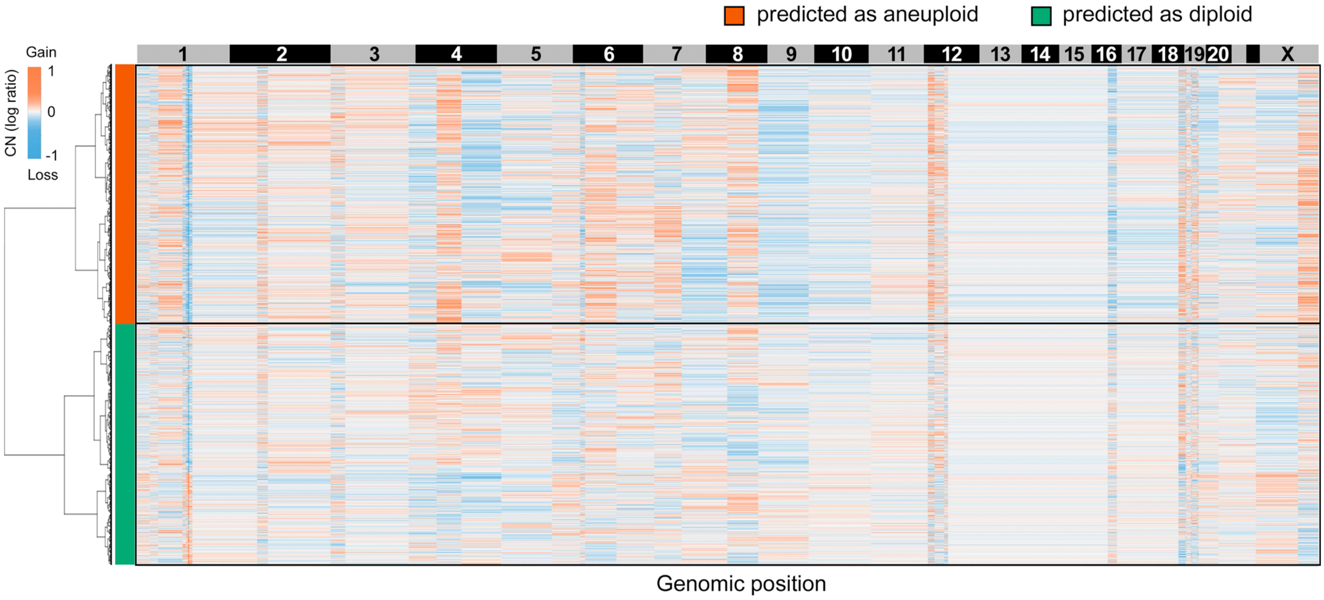

Supplementary Figure 4. Clustered heatmap of single-cell copy number profiles estimated with CopyKAT[36] tool in the AML patient sample.

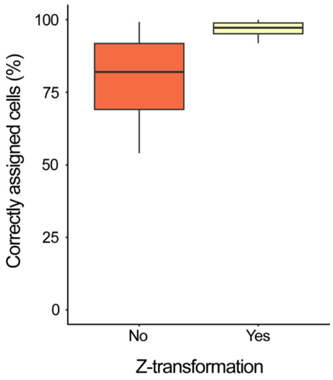

Supplementary Figure 5. The impact of z-score transformation on the ScType annotation accuracy in the 45 simulated datasets with various dropout rates (P<0.001, two-sided Wilcoxon test). The horizontal lines indicate the median, the boxes the interquartile range (IQR), and the whiskers are Q1 - 1.5*IQR and Q3 + 1.5*IQR (where Q1 and Q3 are firsts and third quartiles), across the 45 simulated datasets in each condition.
