# Supplementary material for: Fully-automated and ultra-fast cell-type identification using specific marker combinations from single-cell transcriptomic data
Source: Nat Commun. 2022 Mar 10;13:1246. doi: 10.1038/s41467-022-28803-w (PMC8913782; doi:10.1038/s41467-022-28803-w)
Supplement: Supplementary file 3 — Reporting Summary [file 41467_2022_28803_MOESM3_ESM.pdf]

Corresponding author(s): Anil K. Giri
Last updated by author(s): Nov 20, 2021

Reporting Summary

Nature Portfolio wishes to improve the reproducibility of the work that we publish. This form provides structure for consistency and transparency in reporting. For further information on Nature Portfolio policies, see our Editorial Policies and the Editorial Policy Checklist.

Statistics

For all statistical analyses, confirm that the following items are present in the figure legend, table legend, main text, or Methods section.

n/a	Confirmed	
☐	☒	The exact sample size (*n*) for each experimental group/condition, given as a discrete number and unit of measurement
☒	☐	A statement on whether measurements were taken from distinct samples or whether the same sample was measured repeatedly
☐	☒	The statistical test(s) used AND whether they are one- or two-sided<br>*Only common tests should be described solely by name; describe more complex techniques in the Methods section.*
☐	☒	A description of all covariates tested
☐	☒	A description of any assumptions or corrections, such as tests of normality and adjustment for multiple comparisons
☐	☒	A full description of the statistical parameters including central tendency (e.g. means) or other basic estimates (e.g. regression coefficient) AND variation (e.g. standard deviation) or associated estimates of uncertainty (e.g. confidence intervals)
☐	☒	For null hypothesis testing, the test statistic (e.g. *F*, *t*, *r*) with confidence intervals, effect sizes, degrees of freedom and *P* value noted<br>*Give P values as exact values whenever suitable.*
☒	☐	For Bayesian analysis, information on the choice of priors and Markov chain Monte Carlo settings
☒	☐	For hierarchical and complex designs, identification of the appropriate level for tests and full reporting of outcomes
☒	☐	Estimates of effect sizes (e.g. Cohen's *d*, Pearson's *r*), indicating how they were calculated

Our web collection on statistics for biologists contains articles on many of the points above.

Software and code

Policy information about availability of computer code

Data collection	No software was used
Data analysis	R-packages: Seurat v 4.0.1, scSorter v 0.0.2, SCINA v 1.0.0, scCATCH v 2.1, copykat v 1.0.4 , splatter v 1.16.1

For manuscripts utilizing custom algorithms or software that are central to the research but not yet described in published literature, software must be made available to editors and reviewers. We strongly encourage code deposition in a community repository (e.g. GitHub). See the Nature Portfolio guidelines for submitting code & software for further information.

Data

Policy information about availability of data

All manuscripts must include a data availability statement. This statement should provide the following information, where applicable:

- Accession codes, unique identifiers, or web links for publicly available datasets
- A description of any restrictions on data availability
- For clinical datasets or third party data, please ensure that the statement adheres to our policy

Five datasets were downloaded from Gene Express Omnibus (GEO): Human Liver (GSE124395), Human Brain (GSE67835), Human Pancreas (GSE85241), Mouse Lung (GSE63269) and Mouse Retina (GSE63473). Human PBMC dataset was downloaded from the 10x Genomics Dataset Repository (https://cf.10xgenomics.com/samples/cell/pbmc3k/pbmc3k_filtered_gene_bc_matrices.tar.gz). AML patient sample data were downloaded from the European Genome-phenome Archive (EGA): EGAS00001004614.

The R source-code of the ScType algorithm is freely available at https://github.com/IanevskiAleksandr/sc-type to allow reproduction of the results and its further comparison against or integration with other algorithms. ScType is also freely available as an interactive webtool at http://sctype.app. The ScType database is freely available at https://sctype.app/database.php.

Field-specific reporting

Please select the one below that is the best fit for your research. If you are not sure, read the appropriate sections before making your selection.

☒ Life sciences ☐ Behavioural & social sciences ☐ Ecological, evolutionary & environmental sciences

For a reference copy of the document with all sections, see nature.com/documents/nr-reporting-summary-flat.pdf

Life sciences study design

All studies must disclose on these points even when the disclosure is negative.

Sample size	The development of the ScType method was based on six publicly available scRNA-seq datasets. Each dataset is of various size depending on cell availability in the original experiments ranging from 482 to 44808 cells.
Data exclusions	No data exclusion was done
Replication	No technical replicates were available for testing reproducibility of cell type annotation. However, multiple scRNA-seq datasets were utilized to investigate the performance of ScType and the related methods in the context of various sequencing platforms, tissues types and organisms.
Randomization	Not relevant for this study, as the cell type annotation was done for each sample separately. The study did not involve between group comparisons (e.g. disease vs. control groups).
Blinding	Not relevant for this study, as the cell type annotation was done for each sample separately. The study did not involve between group comparisons (e.g. disease vs. control groups).

Reporting for specific materials, systems and methods

We require information from authors about some types of materials, experimental systems and methods used in many studies. Here, indicate whether each material, system or method listed is relevant to your study. If you are not sure if a list item applies to your research, read the appropriate section before selecting a response.

Materials & experimental systems

n/a	Involved in the study	
☒	☐	Antibodies
☒	☐	Eukaryotic cell lines
☒	☐	Palaeontology and archaeology
☒	☐	Animals and other organisms
☒	☐	Human research participants
☒	☐	Clinical data
☒	☐	Dual use research of concern

Methods

n/a	Involved in the study	
☒	☐	ChIP-seq
☒	☐	Flow cytometry
☒	☐	MRI-based neuroimaging
